# NK Cell-Dependent Growth Inhibition of Lewis Lung Cancer by Yu-Ping-Feng, an Ancient Chinese Herbal Formula

**DOI:** 10.1155/2016/3541283

**Published:** 2016-02-29

**Authors:** Yingbin Luo, Jianchun Wu, Xiaowen Zhu, Chenyuan Gong, Chao Yao, Zhongya Ni, Lixin Wang, Lulu Ni, Yan Li, Shiguo Zhu

**Affiliations:** ^1^Department of Oncology, Shanghai Municipal Hospital of Traditional Chinese Medicine, Shanghai University of Traditional Chinese Medicine, Shanghai 200071, China; ^2^Laboratory of Integrative Medicine, School of Basic Medical Sciences, Shanghai University of Traditional Chinese Medicine, 1200 Cai Lun Road, Shanghai 201203, China; ^3^Department of Immunology and Pathogenic Biology, School of Basic Medical Sciences, Shanghai University of Traditional Chinese Medicine, 1200 Cai Lun Road, Shanghai 201203, China

## Abstract

Little is known about Yu-Ping-Feng (YPF), a typical Chinese herbal decoction, for its antitumor efficacy in non-small-cell lung cancer (NSCLC). Here, we found that YPF significantly inhibited the growth of Lewis lung cancer, prolonged the survival of tumor-bearing mice, promoted NK cell tumor infiltration, increased the population of NK cells in spleen, and enhanced NK cell-mediated killing activity. The growth suppression of tumors by YPF was significantly reversed by the depletion of NK cells. Furthermore, we found that YPF significantly downregulated the expression of TGF-*β*, indoleamine 2,3-dioxygenase, and IL-10 in tumor microenvironment. These results demonstrated that YPF has a NK cell-dependent inhibitory effect on Lewis lung cancer.

## 1. Introduction

The progression of cancer development is decided by a battle between immune system and cancer cells in the body. Due to the genomic instability, cancer cells can escape from immune surveillance to accomplish uncontrolled and fast proliferation. Therefore, the capability of immune escape is an emerging hallmark of cancer cells [1]. The blockade of cancer immune escape or the recovery of cancer immune surveillance should be a promising strategy for cancer immunotherapy [2].

Yu-Ping-Feng (YPF), an ancient Chinese herbal formula, is derived from the Dan-Xi Xin Fa by ZHU Dan-Xi in Yuan Dynasty (A.D. 1279–1368) in China and has been used for the treatment of cold and flu for several centuries in clinical practice. YPF is composed of* Astragali Radix* (Huangqi),* Atractylodis Macrocephalae Rhizoma* (Baizhu), and* Saposhnikoviae Radix* (Fangfeng) in a weight ratio of 2 : 2 : 1. YPF is mainly used for immune regulation [3–5]. For example, YPF can significantly suppress the expression of proinflammatory cytokines in the lipopolysaccharide- (LPS-) induced chronic inflammation model and upregulate phagocytic activity in cultured macrophages through modulating the expression of inducible nitric oxide synthase (iNOS), cyclooxygenase-2 (COX-2), and intestinal alkaline phosphatase (IALP) [6, 7]. YPF can also exert antibacterial and antiviral functions in innate immunity through regulating interferon (IFN) signaling [8].

Non-small-cell lung cancer (NSCLC), accounting for 80% of lung cancer, is the leading death in various cancers all over the world and its 5-year survival rate is less than 5% [9]. Since YPF has a critical modulation effect on immune system and has been used for the treatment of lung cancer [10], we investigated its inhibitory effect on NSCLC through immune regulation. Meanwhile, we also explored the effects of YPF on tumor growth and tumor-bearing mouse survival in a xenograft Lewis lung cancer (LLC) model and evaluated its role in T cell and NK cell tumor infiltration, NK cell cytotoxicity, and the expression of NK cell regulation-associated mediators. Results indicated that YPF had an obvious inhibition on NSCLC through NK cell-dependent regulation. This finding suggests that YPF may be a potent immune regulatory drug for the treatment of NSCLC.

## 2. Materials and Methods

### 2.1. Preparation of YPF

The granules containing* Astragali Radix* (Huangqi),* Atractylodis Macrocephalae Rhizoma* (Baizhu), or* Saposhnikoviae Radix* were purchased from Tianjiang Pharmaceutical Co., Ltd. (Jiangyin, Jiangsu, China), one of six approved manufactures for Chinese herbal granules in China, and mixed well in a mass ratio of 2 : 2 : 1. These granules have been well qualified by HPLC using astragaloside IV, atractylenolide, prim-O-glucosylcimifugin, and 5-O-methylvisammioside as the positive controls according to China Pharmacopoeia 2010 Edition (Supplementary Figure 1 in Supplementary Material available online at http://dx.doi.org/10.1155/2016/3541283).

### 2.2. Reagents

The antibodies (FITC-anti-mouse CD3 (17A2), PE-anti-mouse CD4 (GK1.5), APC-anti-mouse CD8 (53-6.7), APC-anti-mouse NKp46 (29A1.4), purified anti-mouse NK1.1 (PK136)) and murine isotype controls (FITC-IgG1, PE-IgG1, APC–IgG2a, and mouse IgG) were purchased from BioLegend Inc. (San Diego, CA). Calcein-AM was purchased from Sigma-Aldrich (St. Louis, MO).

### 2.3. Animal Procedures

All animal procedures including tumor transplantation, tumor volume measurement, and mouse euthanization were approved by the Institutional Animal Care and Use Committee at Shanghai University of Traditional Chinese Medicine. Lewis lung cancer (LLC) cells were obtained from Shanghai Cell Bank of Chinese Academy of Sciences. The cells were maintained in DMEM medium (Gibco, Grand Ireland, NY, USA) supplemented with 10% fetal bovine serum (FBS), 10% penicillin (100 U/mL), and streptomycin (100 U/mL) (Invitrogen Corporation, California, USA). The male C57BL/6 mice with the age of 6–8 weeks and body weight of 18–20 g were purchased from Shanghai SLAC Laboratory Animal Co., Ltd., and maintained in a pathogen-free environment. The xenografted tumor model was established by subcutaneously inoculating LLC cells (2 × 10^6^ cells in a 50 *µ*L volume per mouse) into the upper back of C57BL/6 mice (*n* = 5) that were preanesthetized with 50 mg/kg of pentobarbital sodium via intraperitoneal injection. The mice were subjected to the intragastric administration of YPF at the daily dose of 116 mg per mouse (equal to 45 g of clinical dose) or the same volume of PBS as the control for 14 consecutive days before the inoculation. For NK cell depletion, 100 *μ*g of PK136 or murine IgG was given via intraperitoneal injection at Day 1 after inoculation and every 3 days thereafter. Tumor size was measured with a caliper every 3 days, and tumor volume was calculated according to the following equation: *V* = (*π*/8)*a* × *b* ^2^, where *V* is tumor volume, *a* is maximum tumor diameter, and *b* is minimum tumor diameter. Mice were sacrificed through CO_2_ suffocation when tumor volume reached up to 2000 mm^3^ or at Day 21 for NK cell depletion assay. Three independent experiments were conducted.

### 2.4. Mononuclear Cell Preparation

Mononuclear cells were isolated from tumor tissue and spleen by smearing the tissue and pushing them through 300 mesh screen twice and then treated with erythrocytolysin. After centrifugation by using mouse percoll (Pharmacia GE), mononuclear cells were collected and applied for intended uses.

### 2.5. Calcein Release Assay

Target cells (LLC cells) were labeled with 2 *μ*g/mL of calcein-AM at 37°C for 1 h during occasional shaking. Effector cells (splenic cells) and target cells were cocultured at the designated effector-to-target (E : T) ratios and incubated at 37°C for 4 h. After incubation, 100 *μ*L of the supernatant was transferred to a new plate. The fluorescence of samples was measured with a Spectramax Gemini EM Fluorescence Microplate Reader (Molecular Devices, CA) (excitation at 485 nm and emission at 538 nm). The lysis rate was calculated according to the equation of [(experimental release − spontaneous release)/(maximum release − spontaneous release)] × 100%.

### 2.6. Flow Cytometric Analysis

Cells were exposed to appropriate antibodies for 30 min at 4°C, washed, and resuspended in PBS containing 1% of FBS. Data were acquired using a FACSCalibur cytometer (BD Biosciences) and analyzed using the FlowJo software (Ashland, OR).

### 2.7. Quantitative Real-Time PCR

Total RNA was extracted from tumor tissues and homogenized in TRIzol reagent (Invitrogen Corporation, California, USA) according to the manufacturer's protocols. qPCR was performed with the PrimeScript*™* RT Master Mix kit (TaKaRa, China) on ABI system (Applied Biosystems, Life Technologies). The PCR protocol included one cycle at 95°C (3 min) followed by 40 cycles of 95°C (15 s) and 55°C (1 min). The expression of the mouse ribosomal 18S mRNA was used as a standard. The primer sequences were as follows: 18S, forward: GTAACCCGTTGAACCCCATT and reverse: CCATCCAATCGGTAGTAGCG; IL-2, forward: CTGAGCAGGATGGAGAATTACA and reverse: AGGTCCAAGTTCATCTTCTAGGC; TGF-*β*, forward: TCTCGATTTTTACCCTGGTGGT and reverse: CTCCCAAGGAAAGGTAGGTGATAGT; indoleamine 2,3-dioxygenase (IDO), forward: CCTGGTTTTGAGGTTTTCGTGTA and reverse: AAGGTTTCAGCATTAAGAAGGTTG; IL-10, forward: AAACAACTCCTTGGAAAACCTCG and reverse: TCCCCAATGGAAACAGCTTAAAC.

### 2.8. Statistical Analysis

All data are expressed as mean ± standard deviation (M ± SD). Tumor volume and survival analysis were evaluated by one-way repeated-measures ANOVA and Mann-Whitney  *U* test, respectively. Others were analyzed by the two-tailed Student's *t*-test. The significant difference was considered at *p* < 0.05.

## 3. Results

### 3.1. YPF Inhibited LLC Tumor Growth and Prolonged Tumor-Bearing Mouse Survival

**Figure 1:**
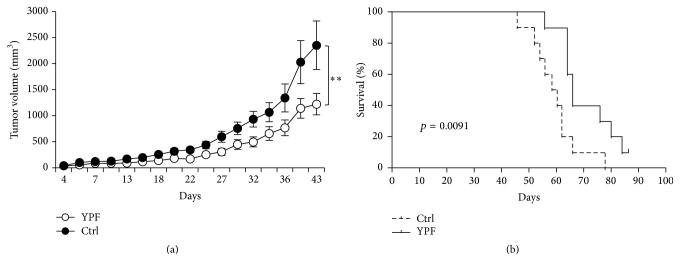
YPF inhibited the growth of LLC tumor and prolonged the survival of tumor-bearing mice. The LLC cells (2 × 10^6^ cells/mouse) were subcutaneously inoculated in the upper back of C57BL/6 mice (*n* = 5). The mice were administered PBS or YPF at the daily dose of 116 mg for each mouse for 14 consecutive days before inoculation. (a) Tumor volume at different time points, ^*∗∗*^ *p* < 0.01; independent experiments were repeated 3 times. (b) Survival curves, *N* = 15, *p* = 0.0091.

In order to determine the effect of YPF on NSCLC, LLC-xenografted mouse model was established. The mice were administered YPF at the daily dose of 116 mg for each mouse by gavage. Results indicated that YPF significantly inhibited the growth of LLC cells when compared to the untreated control (*p* < 0.05) (Figure 1(a)). The median survival time was 66 and 59 days for the LLC-bearing mice with and without YPF treatment and revealed a significant difference (*p* < 0.05) (Figure 1(b)). Therefore, YPF could significantly suppress the growth of LLC tumor and prolonged the survival of LLC-bearing mice.

### 3.2. YPF Increased NK Cell Tumor Infiltration and NK Cell Population in Spleen

**Figure 2:**
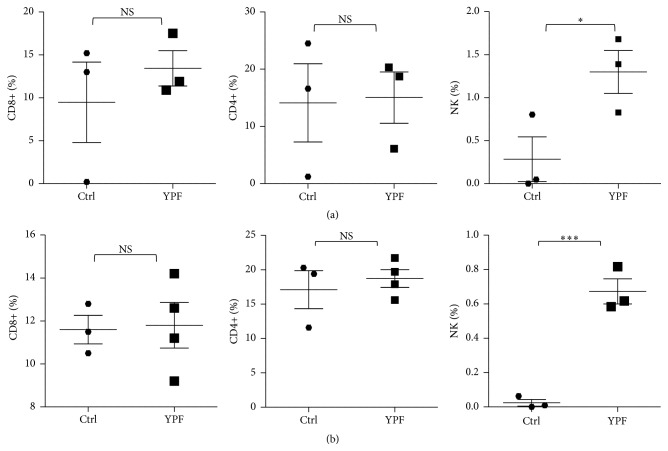
YPF promoted NK cell tumor infiltration and increased NK cell population in spleen. Mononuclear cells were isolated from tumor tissues and spleen of LLC-bearing C57BL/6 mice treated with PBS or YPF at Day 35 after inoculation and then stained with CD3, CD4, CD8, and NKp46, as well as being subsequently analyzed by flow cytometry. (a) Population of CD8+ T, CD4+ T, and NK cells in tumors. (b) Population of CD8+ T, CD4+ T, and NK cells in spleen. ^*∗*^ *p* < 0.05; ^*∗∗∗*^ *p* < 0.001.

Because T and NK cells play an important role in tumor immune surveillance, the effect of YPF on these cells was explored. We analyzed the infiltration of helper T cells (CD3+CD4+), cytotoxic T cells (CD3+CD8+), and NK cells (CD3−NKp46+) in tumor tissue and the population of these cells in spleen by flow cytometry. The results showed that YPF did not induce an obvious change of CD8+ and CD4+ T cells (*p* > 0.05) but significantly increased NK cell tumor infiltration (*p* < 0.05) and NK cell population in spleen (*p* < 0.001) (Figure 2), suggesting that YPF had a positive regulation on NK cells.

### 3.3. YPF Enhanced NK Cell Cytotoxicity

**Figure 3:**
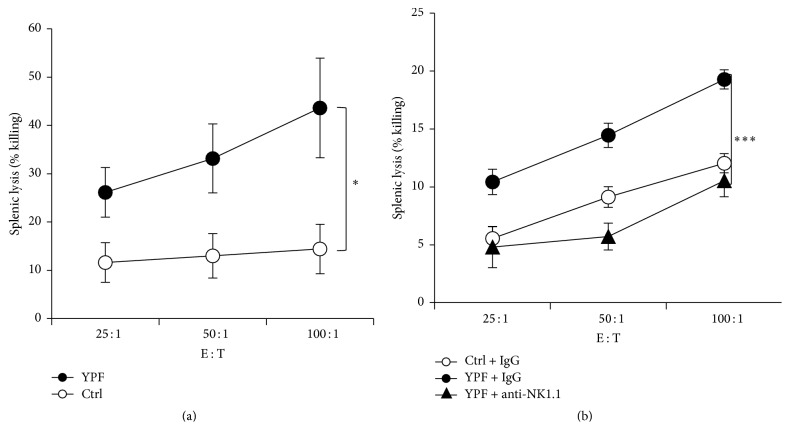
YPF enhanced NK cell-mediated killing activity. Mononuclear lymphocyte cells were isolated from spleens of LLC-bearing C57BL/6 mice treated with PBS or YPFS at Day 35 after inoculation. Target cells (LLC) were stained with calcein-AM and then cocultured with effect cells (splenic cells) at the E-T ratios of 25 : 1, 50 : 1, and 100 : 1. (a) Splenic cells killing activity. (b) Splenic cells killing activity with or without anti-NK1.1 antibody. ^*∗*^ *p* < 0.05; ^*∗∗∗*^ *p* < 0.001.

Due to the enhancement of YPF-induced NK cell tumor infiltration and population in spleen, whether YPF could enhance the cytotoxicity of NK cells is still unclear. Therefore, we isolated splenic cells and evaluated their cytotoxicity through calcein release assay as described in Section 2. The results showed that YPF significantly enhanced the killing activity of splenic cells (Figure 3(a)), which could be reversed by anti-mouse NK1.1 antibody (Figure 3(b)). These results showed that the increase of splenic cell cytotoxicity resulted from the increase of NK cell cytotoxicity, suggesting that YPF could enhance NK cell-mediated killing activity.

### 3.4. NK Cell Depletion Reversed YPF-Mediated Tumor Suppression

**Figure 4:**
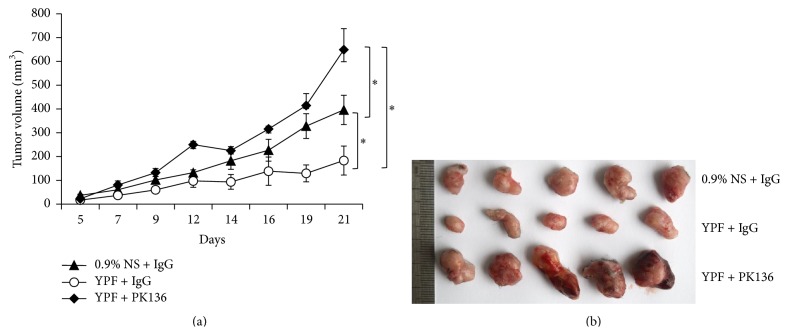
NK cell depletion reversed YPF-induced tumor suppression. LLC-xenografted mouse model was established and treated by YPF as described in Section 2. 100 *μ*g of mouse IgG or PK136 per mouse was administered via IP injection, respectively. (a) Tumor volume at different time points, ^*∗*^ *p* < 0.05; (b) tumor graph at Day 21.

Since YPF significantly increased NK cell tumor infiltration, population, and cytotoxicity, we further explored whether YPF-mediated tumor growth inhibition was dependent on NK cells. The depletion of NK cells was accomplished by using anti-NK1.1 antibody PK136. After the administration of YPF, the inhibition of tumor growth in the presence of YPF was significantly reversed by PK136 antibody (Figures 4(a) and 4(b)), which demonstrated that the suppression of tumor growth by YPF was NK cell-dependent.

### 3.5. YPF Downregulated the Expression of TGF-*β*, IDO, and IL-10

**Figure 5:**
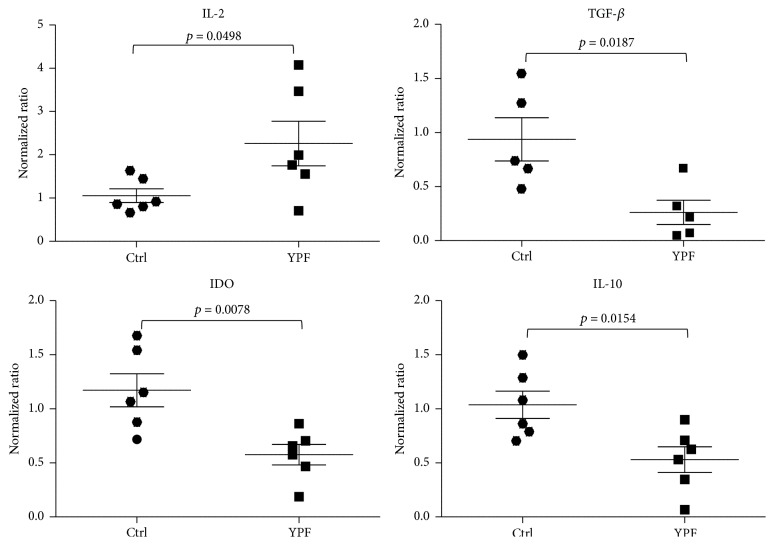
YPF inhibited the expression of TGF-*β*, IDO, and IL-10 in LLC tumors. LLC-bearing mice were treated with PBS or YPF as described in Section 2. At Day 35 after inoculation, total RNA was isolated from tumor tissues and the expressions of IL-2, TGF-*β*, IDO, and IL-10 were analyzed by real-time PCR. The significant difference was considered at *p* < 0.05.

Since the inhibition of tumor growth in the presence of YPF was NK cell-dependent, the exploration of possible mechanisms is highly desired. It is well known that IL-2 is an important regulator for NK cell activation, and TGF-*β*, IDO, and IL-10 are crucial inflammatory mediators for the direct or indirect inhibition of NK cells and the initiation and progression of cancers [11, 12]. We therefore evaluated the expression of these mediators in LLC tumor tissues by real-time PCR. YPF resulted in the increased expression of IL-2 (*p* = 0.0498) but significantly downregulated the expression of TGF-*β*, IDO, and IL-10 (Figure 5), suggesting that YPF can result in the downregulation of TGF-*β*, IDO, and IL-10 in tumor microenvironment.

## 4. Discussion

YPF is an ancient Chinese herbal decoction and has been widely used for the treatment of cold and flu for several centuries and has also been used in cancer patients with idiopathic sweating recently [13]; however, little is known about its antitumor efficacy in NSCLC. In the present study, YPF has been confirmed to significantly inhibit the growth of LLC tumor and prolonged the survival of tumor-bearing mice. YPF can also promote NK cell tumor infiltration, increase NK cell population in spleen, and enhance NK cell-mediated killing activity. The inhibition of tumor growth by YPF can be significantly reversed by NK cell depletion. Further study has demonstrated that YPF can significantly downregulate the expression of TGF-*β*, IDO, and IL-10, thereby directly or indirectly inhibiting the functions of NK cells, which suggests that YPF can suppress NSCLC through NK cell modulation.

NK cells are large granular lymphocytes and play an important role in innate immunity for virus defense and cancer surveillance [14, 15]. Cancer patients usually have low population of NK cells and less NK cell tumor infiltration as well as the heavily impaired NK cell-meditated killing activity [16, 17]. Increasing NK cell tumor infiltration and NK cell-mediated antitumor activity has become a promising strategy for cancer treatment [18, 19]. In this study, YPF can significantly promote NK cell tumor infiltration, increase NK cell population in spleen, and enhance the tumoricidal activity of NK cells. In contrast, the depletion of NK cells can decrease the inhibitory effect of YPF on tumor growth, which provides the convincing evidence that YPF can enhance NK cell function in tumor-bearing mice, and YPF may be a potent immune regulatory drug for cancer immunotherapy.

In tumor microenvironment, lots of mediators produced by cancer cells and cancer-associated stroma or immune cells may impair NK cell activity [18]. TGF-*β* produced by cancer cells, regulator T cells, and tumor-associated fibroblasts can impair NK cell-mediated antitumor activity [20–22]. IDO produced in response to IFN-*γ* in endothelial cells, mesenchymal stromal cells, fibroblasts, and various myeloid-derived cells including dendritic cells and macrophages [23, 24] can suppress NK cell functions [25, 26]. IL-10, an immunosuppressive cytokine, cannot directly inhibit the functions of NK cell effectors but can exert indirect inhibitory effects on NK cell-dampening secretion of IL-12, IL-15, and IL-18 through its accessory cells [11]. In this study, we have found that YPF can downregulate TGF-*β*, IDO, and IL-10 in tumor tissues, which may provide the partial explanation for the YPF-modulated improvement of NK cell function.

In this study, YPF was administered before the tumor establishment and exerted significant tumor growth inhibition; this meant that YPF might have important applications in the prevention of lung tumorigenesis and metastases. Recently, Fei-Liu-Ping, also a Chinese herbal medicine, was shown to inhibit Lewis lung cancer not only for the primary tumor but also for lung metastasis [27]; this suggested that YPF might also have an inhibitory effect on lung metastasis. Of course, additional experiments are needed for further confirmation.

Although YPF has been used for immune regulation for several centuries, it is still the first time to demonstrate the inhibitory effect of YPF on NSCLC through NK cell modulation. All of these findings provide the convincing evidence for its application in lung cancer treatment and extend the application of YPF as a potent immune regulatory drug for the treatment of NSCLC.

## Supplementary Material

The granules containing Astragali Radix (Huangqi), Atractylodis Macrocephalae Rhizoma (Baizhu) or Saposhnikoviae Radix were qualified by HPLC. Astragaloside IV, atractylenolide, prim-O-glucosylcimifugin and 5-O-methylvisammioside were used as the positive controls for Astragali Radix, Atractylodis Macrocephalae Rhizoma and Saposhnikoviae Radix, respectively.
