# Supplementary figures and images for: NK Cell-Dependent Growth Inhibition of Lewis Lung Cancer by Yu-Ping-Feng, an Ancient Chinese Herbal Formula
Source: Mediators Inflamm. 2016 Feb 29;2016:3541283. doi: 10.1155/2016/3541283 (PMC4789500; doi:10.1155/2016/3541283)

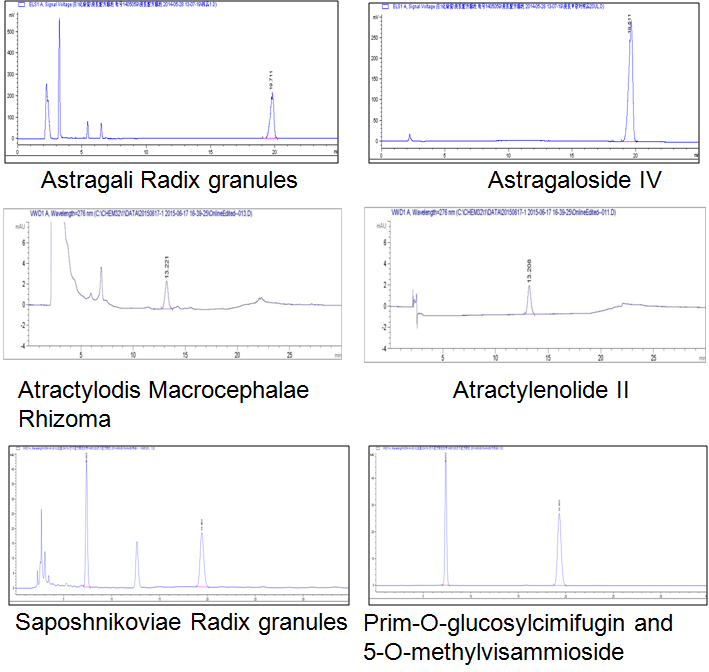

Supplement: Supplementary file 1 — The granules containing Astragali Radix (Huangqi), Atractylodis Macrocephalae Rhizoma (Baizhu) or Saposhnikoviae Radix were qualified by HPLC. Astragaloside IV, atractylenolide, prim-O-glucosylcimifugin and 5-O-methylvisammioside were used as the positive controls for Astragali Radix, Atractylodis Macrocephalae Rhizoma and Saposhnikoviae Radix, respectively. [file 3541283.f1.tif]
